# New perspectives for investigating muscular perfusion response after dietary supplement intake: an exploratory, randomized, double-blind, placebo-controlled crossover trial in healthy young athletes using contrast-enhanced ultrasound (CEUS)

**DOI:** 10.1080/15502783.2022.2097018

**Published:** 2022-07-13

**Authors:** Franziska Bürkle, Julian Doll, Arndt Neide, Simone Gantz, Stefanos Tsitlakidis, Christian Fischer

**Affiliations:** Center for Orthopedics, Trauma Surgery and Spinal Cord Injury, Ultrasound Center, Heidelberg University Hospital, Heidelberg, Germany

**Keywords:** contrast-enhanced ultrasound, CEUS, muscle perfusion, dietary supplements, resistance training, feasibility

## Abstract

**Background:**

Various dietary supplements have been reported to enhance muscular perfusion in athletes practicing resistance training, especially through modulation of nitric oxide signaling.

**Objectives:**

The aim of this study was therefore to investigate selected ‘NO-boosting’ supplements in a real-life setting i) to generate novel hypotheses and perfusion estimates for power calculation in view of a definitive trial and ii) to assess the feasibility of the study design with particular focus on the use of contrast-enhanced ultrasound (CEUS) for perfusion quantification.

**Methods:**

Thirty young male athletes (24 ± 4 years) regularly practicing resistance training were enrolled in this three-arm, placebo(PL)-controlled crossover trial with ingestion of two commercially available supplements: an amino acid combination (AA) (containing 3 g of L-arginine-hydrochloride and 8 g of L-citrulline-malate) and 300 mg of a specific green tea extract (GTE). After intake, CEUS examinations of the dominant biceps brachii muscle were performed under resting conditions and following standardized resistance exercising. Quantitative parameters of biceps perfusion (peak enhancement, PE; wash-in perfusion index, WiPI) and caliber were derived from corresponding CEUS video files. Additionally, subjective muscle pump was determined after exercise.

**Results:**

For PE, WiPI, and biceps caliber, the standard deviation (SD) of the within-subject differences between PL, AA, and GTE was determined, thereby allowing future sample size calculations. No significant differences between PL, AA, and GTE were observed for biceps perfusion, caliber, or muscle pump. When comparing resting with post-exercise measurements, the increase in biceps perfusion significantly correlated with the caliber increase (PE: r = 0.266, p = 0.0113; WiPI: r = 0.269, p = 0.0105). Similarly, the biceps perfusion correlated with muscle pump in the post-exercise conditions (PE: r = 0.354, p = 0.0006; WiPI: r = 0.350, p = 0.0007). A high participant adherence was achieved, and the acquisition of good quality CEUS video files was feasible. No adverse events occurred.

**Conclusion:**

Based on our novel examination protocol, CEUS seems to be feasible following higher-load resistance exercising and may be used as a new method for high-resolution perfusion quantification to investigate the effects of pre-exercise dietary supplementation on muscle perfusion and related muscle size dynamics.

## 1. Introduction

Dietary supplementation among athletes is an important research topic, given that up to 60% of athletes across different sports, sexes, and levels of professionality use supplements [1]. In elite sports, even up to 80% of athletes have been reported to consume dietary supplements, including supplements affecting nitric oxide-mediated signaling [1,2]. Nitric oxide (NO) is an endothelium-derived molecule, which is produced upon activation of the enzyme NO synthase (NOS). NO enhances vasorelaxation by acting on vascular smooth muscle cells [3,4]. In the context of exercise training, this vasorelaxant effect of ‘NO-boosting’ supplements is often mentioned, supporting the hypothesis that these supplements might increase muscular perfusion, resulting in an improved training performance and muscle recovery. However, the efficacy of such ‘NO-boosting’ supplements (e.g. amino acids or plant extracts) is still controversial, due to the lack of evidence for increased blood flow and/or volume after their application [5–11].

An emerging technique to visualize (micro)perfusion and vascularity of various tissues in real time with higher diagnostic accuracy than routine Doppler ultrasound techniques [12–15] is contrast-enhanced ultrasound (CEUS). CEUS is gaining importance in the field of musculoskeletal medicine and sports sciences [16]. CEUS has an exceptionally favorable safety profile, being based on the ultrasound contrast agent (UCA) SonoVue® (Bracco, Milan, Italy) [16–18]. With the latter remaining purely intravascular [19], CEUS allows safe and repetitive quantification of muscular microperfusion. To ensure reliability of measurements, standardized algorithms have been used, which imply strict avoidance of out-of-plane movements by the examined subject relative to the ultrasound probe [16,20]. To our knowledge, muscular microperfusion in response to acute resistance training after supplement intake has never been addressed so far via CEUS. Nevertheless, CEUS has been used to investigate numerous perfusion-related processes and pathologies of the skeletal muscles [21–26]. While some of the studies investigated the immediate effects in subjects exposed to acute muscle activation by low-load exercise prior to CEUS [21–23], others investigated late-onset effects after several hours of applying loads up to 25% of the subjects’ body weight [24]. Since the operating principle of quantitative CEUS as applied in our study is to extract blood flow parameters from a specific predefined scan plane, an essential task of the examiner is to ensure a stable transducer position using current guideline recommendations [16,27,28]. Performing CEUS immediately after higher-load resistance training would therefore represent a new challenge for practical implementation, considering the increase in respiratory body movement linked to higher cardiovascular exertion in comparison with the aforementioned study settings.

As so far only limited evidence regarding the effects of NO-boosting dietary supplements on muscle perfusion is available, we wanted to investigate this in further detail using CEUS. Our exploratory study will provide important information for planning a future main trial with more patients. The primary outcomes were i) to examine selected commercially available NO-boosting supplements in young athletes using CEUS in an explorative way, including estimates of the muscular perfusion increase for power calculations and ii) to assess the feasibility of the CEUS-based study protocol in a small study cohort.

Secondary outcomes were the correlation of muscle size parameters with quantitative CEUS perfusion results.

## 2. Methods

### Trial design and recruitment

The trial design was set up as described previously [29]. Briefly, we used the CEUS bolus technique followed by perfusion quantification to compare muscular perfusion within a standardized scan plane under the influence of different pre-workout supplements in a placebo-controlled crossover design. This study was conducted at a DEGUM (German Society of Ultrasound in Medicine)-accredited university ultrasound center affiliated with a center for orthopedics and trauma surgery. The study was prospectively registered at the German Clinical Trials Register (DRKS00016972). Recruitment of volunteers was carried out through advertisements in university departments and fitness centers. Volunteers who were interested in participation were invited to a first appointment, including an eligibility screening with health status assessment (e.g. medical history regarding previous diseases, medication, and supplement intake in the past). Young male recreational athletes (age ≥ 18 years) regularly practicing resistance training were included in the study after obtaining informed consent. Full exclusion criteria are provided in Table S1 [see Additional File 1]. This study being an exploratory trial, we intended to include 30 subjects [30].

### Interventions

Study interventions consisted of three different pre-exercise drinks, containing i) an amino acid combination (AA) of 3 g of L-arginine hydrochloride (0.83 g L-arginine/g L-arginine-hydrochloride) with 8 g of L-citrulline-malate (0.7 g of L-citrulline/g L-citrulline-malate) (Elite Sports Nutrients, Fitmart, Elmshorn, Germany) dissolved in 200 mL of water, ii) 300 mg of a specific green tea extract (GTE) (Vaso6, Serious Nutrition Solutions, Danville, VA, USA) dissolved in 200 mL of water, or iii) only 200 mL of water as placebo control (PL).

For blinding purposes, 1.5 g of citric acid was added to the GTE and PL treatments. To simulate real-life conditions of supplement use, supplement dosing was chosen according to the recommendations of the manufacturer. These doses were previously reported to be safe and well tolerated for pharmaceutical products comparable to the supplements used in our study [31–34]. Timing of the main perfusion measurement was determined based on the available pharmacokinetic data, demonstrating maximum plasma L-arginine levels 60 minutes after intake [35]. The L-citrulline-malate dose was chosen based on saturation effects observed at 15 g, and hence, lower dosages were proposed for practical use [31]. To ensure double-blinding, an external statistician generated the allocation schedule by computer. Thus, subjects were assigned pseudo-randomly (counterbalanced) to one of the six treatment sequences of the Williams design. All participants had to complete three sessions, each separated by a minimum of 7 days of washout. Subjects were instructed to maintain their weekly training routine throughout the study and to refrain from any supplement intake other than the study interventions.

### Experimental day protocol

**Figure 1.**
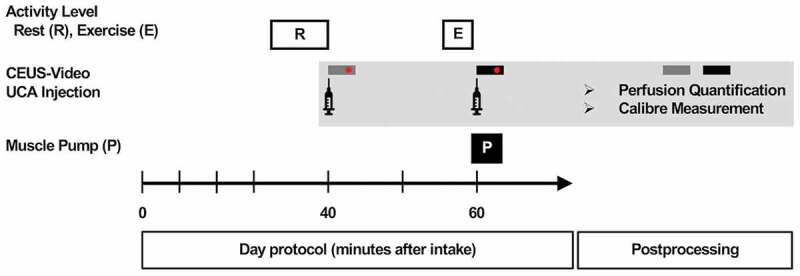
Experimental day and video postprocessing protocol. Each study session included two CEUS examinations following different levels of activity. Specified times refer to the initial supplement intake (minute 0). The CEUS examination after 40 minutes was preceded by controlled resting in the supine position. For biceps muscle activation, subjects had to complete standardized resistance exercising according to the protocol. Sixty seconds after completion, the second CEUS examination was performed, in total 60 minutes after supplement intake. Muscle pump was determined immediately after exercising. CEUS videos under resting (gray bar) and post-exercise (black bar) conditions were then analyzed regarding perfusion quantification and caliber measurements. UCA, ultrasound contrast agent.

All appointments were set up in the morning, and subjects reported to the ultrasound center after an 8-hour fasting period. Forty minutes after supplement intake, the first CEUS examination was performed under resting conditions (Figure 1). Subsequent workout was supervised by the same blinded physical therapist for all appointments. Warm-up included 20 concentric-eccentric biceps brachii muscle contractions (biceps curls) of the dominant arm with the weight of the dumbbell bar only, , which was 1.8kg. This was also useful to familiarize study participants with the final exercise for biceps muscle activation. Maximum isometric strength (MIS) was determined during maximal isometric contraction of the dominant biceps muscle in the neutral position with 90° flexion of the elbow and forearm supination for three seconds using a handheld dynamometer. Both warm-up and MIS determination procedures were performed at each of the three study sessions due to the concomitant activation of muscle fibers and muscular perfusion. The weight of the adjustable dumbbell was adapted to values around the originally planned weight of 30% of the maximum isometric strength (corresponding to ~55% 1RM [36]) in consideration of the subjects’ training status, which was accounted for in the statistical analysis. Finally, biceps muscle activation was based on common hypertrophy-oriented training recommendations [37,38]: starting from the neutral position, subjects had to complete 4 sets of 10 dumbbell biceps curls using 1 s for concentric and 1 s for eccentric movement in each repetition (resulting in a time under tension of 20 s for each set). Resting pauses of 60 s between sets were monitored by means of a stopwatch. One minute after completion of the last set, which in total was 60 minutes after supplement intake, a second CEUS examination was performed to determine muscular perfusion under post-exercise conditions.

### Measurements

#### CEUS procedure

The CEUS examination protocol was applied in accordance with recently published guidelines for musculoskeletal applications [16]. All examinations were performed using an ACUSON S2000 ultrasound device (Siemens Healthineers) in a darkened and temperature-controlled room, with the subjects in supine position, while their dominant arm was fixed in 70° abduction and full supination on a positioning pillow. A linear probe (4-9 MHz) was used to transversally adjust the standardized scan plane at 2/3 the distance from the anterior axillary line to the medial epicondyle of the humerus [39].

The applied algorithm for safe probe handling and scan plane adjustment is provided online [see Fig. S1, Additional File 2]. This allowed a reproducible B-mode visualization of the biceps muscle cross-section. Without changing the probe position, the live dual view (Contrast Cadence and B-mode) was applied for subsequent CEUS measurements with the following technical settings: a gain of 3 dB, a dynamic range of 80 dB, and a mechanical index (MI) of 0.1-0.14 (adjusted within this small range for image optimization). A 2.4-mL UCA bolus was injected intravenously and flushed with 10 mL of 0.9% saline solution. Starting from the time point of injection, a 70-s video clip (frame rate 5 Hz) of the corresponding contrast agent wash-in was recorded in the standardized scan plane for post hoc perfusion quantification.

#### Quantification

**Figure 2.**
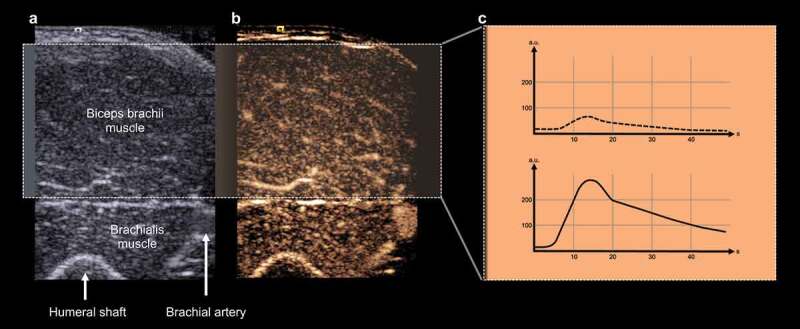
Standardized scan plane for contrast-enhanced ultrasound examination of the biceps muscle tissue. (a) The B-mode image visualizes the humeral shaft and the brachial artery cross-sections as important landmarks for scan plane assessment. Based on the highlighted area as illustrated in the corresponding contrast mode image (b), (c) time intensity curves are generated during UCA bolus passage under resting conditions (dotted line) and following standardized resistance exercising (solid line), reflecting biceps muscle perfusion. a.u., arbitrary units.

Perfusion was quantified using the designated VueBox software (v 7.1; Bracco Imaging). One region of interest (ROI) was chosen in the recorded video clip, including the visualized biceps muscle cross-section. Fasciae and larger arteries causing distorting signals were excluded. Time intensity curves (TICs) were generated from the changing signal intensities in the ROI during UCA bolus passage [27,28], and perfusion assessment was quantified in arbitrary units (a.u.) based on the following parameters (Figure 2) [19]:

•Peak enhancement (PE): the maximum signal intensity of the enhancement curve, describing the maximum blood volume in the ROI

•Wash-in perfusion index (WiPI): reflecting the blood inflow per time until peak enhancement, calculated as the ratio between the area under the curve and the wash-in duration of the UCA in seconds

The quantified biceps perfusion under resting conditions was used as a baseline. The tested supplements advertised as ‘boosters’ [40] are suggested to predominantly exert their effect in the context of exercise, which itself is associated with activation of endothelial NO synthase (eNOS) [41–43]. Therefore, the influence of the different supplements on perfusion values under resting conditions was considered to be negligible.

#### CEUS reliability

All CEUS examinations were performed by the same orthopedic and trauma consultant with DEGUM level III (German Society for Ultrasound in Medicine) qualification (C.F.), who was strictly blinded to any treatment.

To ensure within-subject comparability across all perfusion measurements, we used a standardized algorithm combined with a screenshot taken during the first examination. The latter should allow precise matching of all adjusted scan planes throughout the study under consideration of subject-specific image characteristics. Moreover, the settings from the participants’ first CEUS examination were applied for any follow-up measurements (e.g. depth and focal depth, both depending on the respective biceps caliber).

In our preliminary study on muscular CEUS, we describe that sectional plane concordance is a major determinant of reproducibility, with close-to-perfect intraclass correlation coefficients (ICC) and low coefficients of variation (CV) [20]. Accordingly, we defined parameters for the assessment of sectional plane concordance in this study. The first parameter (P1), based on a cadaveric study by Buranaphatthana et al. [44], demonstrates that, depending on the measurement location relative to the longitudinal humeral axis, the brachial artery may have differing distances from the humerus in the mediolateral direction. As the standardized scan plane for CEUS examination of the biceps brachii muscle includes both, the humerus and the brachial artery cross-sections, the latter can be used to detect intraindividual sectional plane variations between the appointments.

Accordingly, in all CEUS videos from the resting measurements, the distance between the center of the brachial artery and the humerus was assessed, using the muscle–bone interface of the forearm flexor muscle thickness measurement as described by Jenkins et al. [45]. The forearm flexor muscle thickness itself was used as a second parameter (P2). Together, these parameters primarily allowed the assessment of i) varying transducer pressures (leading to variably restricted blood flow) and ii) sectional plane variations relative to the longitudinal humeral axis (leading to imaging of histologically different muscle slices). These two factors could lead to a major bias in comparisons, if not well controlled for.

#### Muscle size dynamics

Muscle size dynamics were evaluated via biceps caliber measurement (under resting and postexercise conditions) as well as the subjective ‘muscle pump’ score (after exercise). For biceps caliber measurements, CEUS video clips were processed as follows: the B-mode image acquired prior to UCA arrival was used to perform three caliber measurements of the biceps muscle between the external and internal fascia, determined by three vertical imaginary axes passing through i) the center of the humerus, ii) the lateral wall of the brachial artery, and iii) the center of the distance in between.

The calculated means under resting and postexercise conditions were used for further analyses. Moreover, subjects had to rate their feeling of muscle pump perceived directly after exercising, also referred to as ‘cellular swelling’ together with hyperemia [46], on a visual analog scale from 0 to 10 (0 ‘no muscle pump at all’ and 10 ‘maximum conceivable muscle pump’).

#### Dietary intake

Dietary intake between 6:00 P.M. and the beginning of the 8-hour fasting period before the first examination was individually documented on paper by the study participants. They were then instructed to ingest meals with a similar nutrient composition prior to the second and third appointments, which was again documented. Thus, adherence to the protocol was verified regarding similar presleep macronutrient ingestion, and its influence on baseline blood flow measured by CEUS is unknown so far. However, presleep macronutrient ingestion has been shown to alter morning metabolism and muscle protein synthesis [47,48], thereby harboring a risk of inconsistently affecting muscle perfusion [49].

The obtained data were analyzed using the NutriGuide software (v 4.8) for total values of carbohydrate (g), fat (g), protein (g), and energy (KJ).

### Statistical methods

Characteristics and dietary data of study participants are presented descriptively (Table 1). Additionally, dietary data were compared between the different treatment groups using the Friedman test.

**Table 1.** Subject characteristics in total and presleep dietary data by treatment.

		Total study population	
Age (yrs)		24 (4)	
Height (cm)		180.1 (5.9)	
Weight (kg)		82.3 (6.6)	
Experience of resistance training (yrs)†		6 (3)	
Training frequency (per week)*		4 (2-7)	
Biceps curl MIS (kg)		26.5 (3.4)	
	**Group PL**	**Group GTE**	**Group AA**
Carbohydrates (g)	99.2 (74.0)	94.4 (69.7)	98.3 (83.3)
Fat (g)	28.9 (33.0)	32.2 (28.3)	33.5 (39.3)
Protein (g)	49.7 (44.5)	59.2 (59.4)	60.7 (54.0)
Energy (KJ)	3638.9 (2593.6)	3863.5 (2732.8)	3985.3 (3074.1)

Data are means (standard deviation) based on n = 30 except where indicated.

No statistically significant difference between treatments for presleep dietary data (p > 0.05 for all comparisons). MIS, maximum isometric strength; PL, placebo; GTE, green tea extract; AA, amino acid combination

† n = 29

* Median (range)

Subject characteristics by sequence group including resting estimates of main outcomes averaged over periods can be found in Table S2 (see Additional File 3).

Given the physiological variation in resting muscular blood flow [50] and corresponding CEUS quantification data, which are known to correlate with relevant venous blood parameters including the plasmatic intravascular volume fraction [51], all calculations for treatment comparison were adjusted for baseline values.

To allow sample size calculation for a future main trial, CEUS perfusion estimates as well as caliber outcomes were reported together with their 95% confidence interval (CI) and standard deviation (SD) of within-subject differences between the treatment groups.

For perfusion and biceps caliber analysis, an ANCOVA was conducted accounting for age and the dumbbell weight relative to the MIS as covariates, following recommendations in the literature [52]. Outcomes are reported as point estimates and 95% CIs.

Moreover, pump scores under different treatment conditions were compared following a nonparametric approach adjusting for period effects as described by Senn [53]. To determine point estimates and 95% CIs, a median-scaling concept was applied for each pairwise treatment comparison, including sequence stratification and the Hodges Lehmann estimator [54]. Reported p-values are two-sided. The level of significance was set at ≤0.05. Due to the exploratory nature of this study and therefore small study sample size, no adjustments were made for multiple comparisons.

Associations between perfusion, caliber, and muscle pump were analyzed using Pearson and Spearman correlation coefficients.

Statistical analyses were conducted using SAS for Mac OS X (*SASUniversityEdition*), while for graphic visualization, Prism software (v 8.4.3; GraphPad Software) was used.

## 3. Results

### Participant flow and baseline characteristics

**Figure 3.**
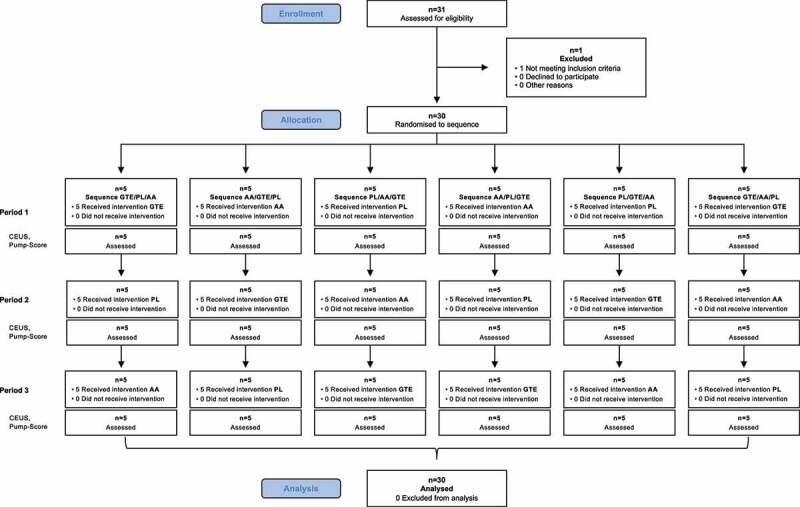
Consolidated Standards of Reporting Trials (CONSORT) flow diagram. PL, placebo; GTE, green tea extract; AA, amino acid combination.

Out of 31 subjects who were screened for eligibility, 30 subjects were randomized, completed the trial, and were included in the final analysis. The CONSORT participant flow throughout the study is shown in Figure 3. Patient enrollment started in August 2019, and follow-up was completed in September 2020. The included subjects were young (mean 24 ± 4 years), male and regularly practiced resistance training (mean training frequency per week: 4, range 2-7). Subject characteristics and pre-sleep dietary data preceding the appointment, which did not differ significantly between treatments, are presented in Table 1.

### Outcomes

No adverse events occurred related to UCA application, and all 180 CEUS video files (with out-of-plane movement as a predetermined exclusion criterion) could be included for analysis.

Excellent ICCs were found with regard to sectional plane concordance as defined by the two parameters described in the Methods section (P1: r = 0.995, 95% CI 0.992, 0.998; P2: r = 0.995, 95% CI 0.991, 0.997).

The estimates of PE and WiPI perfusion increase (postexercise minus resting estimates) by treatments are displayed in Table 2, together with underlying condition estimates.

**Table 2.** Changes from resting to postexercise conditions for main outcomes with underlying estimates by treatment.

	Resting estimates			Postexercise estimates			Changes (postexercise minus resting estimates)†		
	PL	GTE	AA	PL	GTE	AA	PL	GTE	AA
PE (a.u.)	10.50 (10.78)	11.00 (7.60)	10.28 (6.23)	258.11 (106.41)	280.14 (110.72)	262.90 (108.35)	247.61 (100.36)	269.15 (106.84)	252.62 (106.70)
							[210.14, 285.09]	[229.25, 309.04]	[212.78, 292.46]
WiPI (a.u.)	6.83 (7.00)	7.19 (4.95)	6.70 (3.97)	161.96 (66.66)	175.95 (69.46)	164.61 (67.50)	155.13 (62.75)	168.76 (66.92)	157.91 (66.44)
							[131.70, 178.56]	[143.77, 193.75]	[133.10, 182.72]
Caliber (cm)	2.55 (0.40)	2.56 (0.36)	2.48 (0.39)	2.87 (0.50)	2.87 (0.47)	2.84 (0.49)	0.32 (0.16)	0.31 (0.20)	0.36 (0.19)
							[0.25, 0.38]	[0.24, 0.39]	[0.29, 0.43]

Data are means (standard deviation) based on n = 30; PE, peak enhancement; WiPI, wash-in perfusion index; (a.u.), arbitrary units; caliber, biceps caliber; PL, placebo; GTE, green tea extract; AA, amino acid combination

†Additionally provided: [95% CI]

Considering the parameter of PE, the SD of the within-subject differences between PL and GTE (PL minus GTE) was −0.219 a.u. (95% CI −0.6, 0.1); for comparison between GTE and AA (GTE minus AA), the SD was 0.153 a.u. (95% CI −0.2, 0.5); and between PL and AA (PL minus AA), the SD was −0.048 a.u. (95% CI −0.4, 0.3). Regarding WiPI, the SD of the within-subject differences between PL and GTE (PL minus GTE) was −0.221 a.u. (95% CI −0.6, 0.1), while for comparison between GTE and AA (GTE minus AA), the SD was 0.159 a.u. (95% CI −0.2, 0.5) and between PL and AA (PL minus AA), the SD was −0.042a.u. (95% CI −0.4, 0.3).

**Figure 4.**
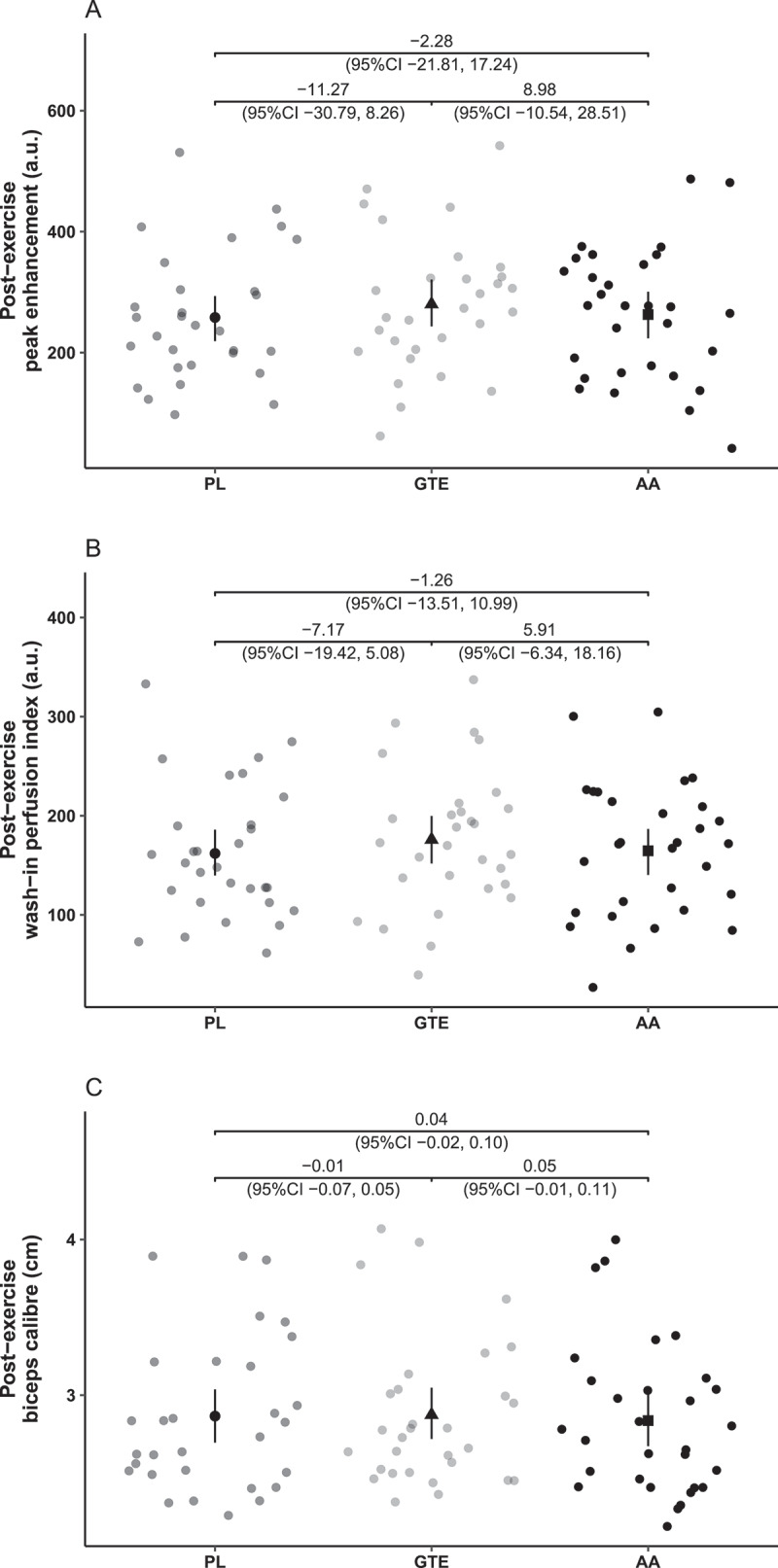
Treatment comparison of biceps perfusion and caliber. Panel a represents the CEUS perfusion parameter of peak enhancement, and panel b the CEUS perfusion parameter of wash-in perfusion index. Panel c represents the biceps caliber as a parameter of muscle size dynamics. For treatment comparison, point estimates and 95% CI from a linear mixed model accounting for age and dumbbell weight relative to the MIS are shown together with underlying data. PL, placebo; GTE, green tea extract; AA, amino acid combination; (a.u.), arbitrary units; MIS, maximum isometric strength.

When comparing adjusted perfusion estimates of PE and WiPI between treatments, no significant differences could be detected (Figure 4(a,b)).

### Secondary outcomes

Besides perfusion outcomes, Table 2 summarizes estimates of caliber changes (postexercise minus resting estimates) and underlying condition estimates.

Regarding biceps caliber, the SD of the within-subject differences between PL and GTE (PL minus GTE) was 0.026 cm (95% CI −0.3, 0.4). When comparing GTE and AA (GTE minus AA), the SD of the within-subject differences was −0.212 cm (95% CI −0.6, 0.1), and when comparing PL and AA (PL minus AA), it was −0.197 cm (95% CI −0.6, 0.2).

Consistent with microperfusion-related findings, biceps caliber (Figure 4(c)) and muscle pump (Table 3) did not show any significant differences between treatments.

**Table 3.** Treatment comparison of muscle pump.

	PL vs. AA	AA vs. GTE	PL vs. GTE
Muscle pump	0.5	−0.25	0
	[0.00, 1.00]	[−0.75, 0.00]	[−0.50, 0.50]

Data are Hodges-Lehmann point estimates and 95% CI for treatment differences after median-scaling. PL, placebo; GTE, green tea extract; AA, amino acid combination

As expected, the model used for PE, WiPI, and biceps caliber analysis showed resting values to increase significantly after exercise regardless of treatments (p < 0.0001 for all variables).

Under resting conditions, there were no significant correlations between CEUS perfusion parameters and biceps caliber. However, the absolute exercise-induced increases in CEUS perfusion parameters and biceps caliber correlated significantly (PE: r = 0.266, p = 0.0113; WiPI: r = 0.269, p = 0.0105). Moreover, the CEUS perfusion parameters after exercise significantly correlated with muscle pump (PE: r = 0.354, p = 0.0006; WiPI: r = 0.350, p = 0.0007).

## 4. Discussion

In this study, CEUS was used to investigate muscular microperfusion after oral intake of NO-boosting supplements in response to acute resistance training in healthy young athletes.

To our knowledge, this is the first study to address this question using CEUS. CEUS application in this setting was safe, as no adverse events occurred. Furthermore, the risk of out-of-plane movement was well controllable using the algorithm described in this study, thereby allowing the CEUS-based acquisition of quantitative data.

Sectional plane concordance (according to the above mentioned definition), which has previously been described to be a crucial prerequisite for reproducibility of perfusion measurements [20], was also given in the current setting when comparing different periods.

Regarding the main outcome variables, the SDs of the within-subject differences between the treatment groups were reported together with 95% confidence intervals. Thus, these data can allow sample size calculations as described previously [55,56].

Due to insufficient power of this study to assess differences between treatment groups (limited sample size) [Table S3, see Additional File 4], results should be interpreted carefully. In our study, adjusted estimates of CEUS perfusion parameters did not differ significantly between treatments nor did the secondary outcomes of muscle size dynamics, which may reflect our small sample size.

The potential mechanisms and clinical relevance of NO-boosting nutrients are still controversial. Our exploratory study, however, will allow for the design of a larger, well-powered CEUS-based study addressing this question in further detail.

### Measuring microperfusion under amino acid-based supplementation

For AA treatment, we combined common L-arginine with L-citrulline. The combined ingestion of both amino acids has previously been shown to increase plasma L-arginine levels more effectively than mere L-arginine supplementation [35,57]. Moreover, enhanced NO production and blood circulation were observed, which was attributed to improved substrate availability for eNOS [57]. However, in our study cohort, muscular microperfusion did not increase under AA compared to the other treatment groups. This may in part be explained by the so-called “L-arginine paradox.” Comparing the Michaelis constant (Km) of eNOS in vitro with the relatively high physiological plasma L-arginine concentrations, eNOS might already be saturated under normal conditions. Thus, exogenous supplementation to increase L-arginine levels might not further promote NO synthesis and thereby vasodilation in humans. However, the latter is still a matter of debate [58–60].

Asymmetric dimethyl-arginine (ADMA), an endogenous competitive NOS inhibitor, has been shown to be elevated in patients with cardiovascular diseases [61,62]. Exogenous L-arginine may therefore be helpful in restoring NOS activity in this patient subset, thereby promoting endothelium-dependent vasodilation [58]. Similarly, Schwedhelm et al. justified their negative results regarding the effects of L-arginine or L-citrulline supplementation on flow-mediated vasodilation in a non-clinical setting [61]. This study involved healthy subjects with normal ADMA concentrations and intact vascular function. Although ADMA levels were not assessed in our study cohort, we assume that ADMA levels were within the normal range, since only healthy young athletes without vascular diseases were included.

### Measuring microperfusion under green tea-based supplementation

Under GTE treatment, no significant improvements in muscular microperfusion were found upon comparison with other treatment groups. Within procyanidin-rich natural extracts, oligomeric and galloylated forms of procyanidins have been identified as important mediators of the clinically relevant effects observed in experimental studies, such as endothelium-dependent relaxing (EDR) activity in the rat aorta [63–66]. The GTE product used in this study is advertised as containing such potent procyanidins, the latter belonging to the large group of polyphenols [67–70]. According to Spencer et al. [71], the metabolite profile after polyphenol intake depends on the ingested dose, with free forms occurring in human plasma after intake of high pharmacological doses but not after lower dietary quantities. Moreover, it needs to be determined to which extent different conjugates of procyanidins are clinically effective as compared to in vitro studies based on free forms of procyanidins [72]. A recent exploratory study demonstrated increased localized postexercise blood flow after ingestion of a 600 mg dose of the investigated green tea-based supplement, however, not after a 300 mg dose (consistent with our findings) [10].

In this study, commercially available products were used to simulate real-life conditions of dietary supplementation. Previous work found substantial variability of supplement quality among commercial products, where chemical analysis showed, e.g., lower ratios of citrulline to malate than was indicated on the product labels [73,74]. Thus, given that we did not analyze the actual content of tested supplements, product characteristics leading to low plasma levels of active compounds cannot be fully excluded.

In summary, the exact determination of the dietary product composition, ADMA plasma levels as well as the additional use of higher GTE doses should be considered when planning a follow-up study with a larger sample size. This should then allow a comprehensive interpretation of perfusion results, along with practice-oriented recommendations.

### Link between muscle size dynamics and microperfusion with implications for training optimization

As a parameter of muscle size dynamics, biceps caliber was measured as previously mentioned. Based on another trial with a similar exercise protocol, this site on the biceps’ longitudinal axis was selected, as it has been shown to display the most pronounced and consistent swelling immediately postexercise compared to the control across visits [39]. In this trial, biceps caliber significantly increased after exercise, which was also observed for CEUS perfusion parameters. Dynamic exercise is known to promote vasodilation in the active muscle [75], which was related to increases of intramuscular water content through fluid shifts [76,77]. In line with this, another study conducted by Ploutz et al. found acute decreases in plasma volume after exercising to be associated with increases of active muscles’ cross-sectional area [78].

In our study, the link between elevated blood flow and the muscle size in response to exercise may be reflected by the weak but significant correlation between absolute changes in microperfusion and biceps caliber throughout exercising. Muscular microperfusion may represent only one factor among others to determine the degree of fluid shift [79], although it appears to play a relevant role as such. This assumption is additionally supported by our observation of a significant correlation between postexercise microperfusion and the specified muscle pump, which subjectively estimates the resulting pooling of fluid within the muscle and its cells, respectively [46]. Cell swelling, together with its favorable effects on protein accretion, is hypothesized to mediate some of the hypertrophic muscular response to training via involvement of osmosensors and subsequent activation of anabolic signaling pathways [38,46,80]. For hypertrophy-oriented resistance training as investigated in this study, methods investigating acute mechanisms related to cell swelling such as CEUS might be helpful to better understand the process of long-term anabolic adaptations in trained muscles.

### Limitations and strengths

Maximum strength was measured isometrically using a hand grip dynamometer. The latter is routinely used in musculoskeletal CEUS [16] as it is easy to use, time-saving, and broadly applicable even in a clinical setting with elderly and preoperated patients [21,36,81]. Nevertheless, in the case of young healthy subjects, determination of maximal strength using the current gold standard (1RM testing or 3-5RM testing to predict a 1-RM value using a dumbbell) during an additional appointment might have been beneficial, as it is more specific to the isotonic biceps curl.

The consumption of alcohol (beyond the 8 hours of fasting together with the preparation time on site) was not an exclusion criterion in our study, although the latter might affect our study results considering the observed hangover-related augmentation of cardiac output [82].

Two key parameters of sectional plane concordance were assessed, including muscle thickness as a parameter of varying transducer pressure. However, muscle thickness is poorly sensitive to tilt of the ultrasound probe [83], which may affect echogenicity. In our study, we only controlled for probe tilt by strict adherence to the examination protocol. Future studies could additionally use a small electronic level with a digital readout analogous to Dankel et al. [83], attached to the probe, to further control for possible effects of tilting, especially in the case of little sonographer experience.

The primary focus of this exploratory trial was to investigate the diagnostic value of CEUS for nutrition-related examination of microperfusion. Furthermore, our exploratory study might allow the power calculation for a future main study in a real-life setting of supplement use.

Therefore, plasma levels or ingredient composition of tested supplements were not investigated in detail.

In general, laboratory testing prior to CEUS examination is not required, as this contrast agent is eliminated through the airways within minutes after administration [16,84,85]. In addition to its favorable safety profile, CEUS allows us to detect quantitative changes of microperfusion and caliber at the same time with high resolution.

## 5. Conclusion

This study investigated alterations in muscle microperfusion after acute resistance training upon ingestion of commercially available NO-boosting supplements using CEUS. Taken together, our study demonstrates that CEUS is feasible also in settings with higher-load resistance training. A standardized algorithm for safe probe handling and scan plane adjustment was developed, allowing the investigation of changes in muscular microperfusion and muscle size dynamics in the context of dietary supplements and/or specific training regimens.

## Supplementary Material

Supplemental Material

Supplemental Material

Supplemental Material

Supplemental Material

## Data Availability

The data supporting the findings of this trial are provided by the corresponding author upon reasonable request.
