# Supplementary material for: New perspectives for investigating muscular perfusion response after dietary supplement intake: an exploratory, randomized, double-blind, placebo-controlled crossover trial in healthy young athletes using contrast-enhanced ultrasound (CEUS)
Source: J Int Soc Sports Nutr. 2022 Jul 13;19(1):397–416. doi: 10.1080/15502783.2022.2097018 (PMC9291664; doi:10.1080/15502783.2022.2097018)
Supplement: Supplemental Material [file RSSN_A_2097018_SM0845.pdf]

1. PROBE POSITIONING

- at the transition between the middle and distal third of the distance from the anterior axillary line to the medial epicondyle of the humerus
- with the elbows fixed on a solid surface for stability of the positioned probe

↓

2. PROBE ADJUSTMENT

- orthogonal to the biceps' longitudinal axis

↓

3. SCAN PLANE ASSESSMENT

based on the following landmarks:

- the humeral shaft and the brachial artery cross sections, which bound the displayed image on both sides
- the horizontally visualised fascia separating biceps and brachialis muscle bellies
